# Exploring the role of *Mycobacterium avium* subspecies *paratuberculosis* in the pathogenesis of type 1 diabetes mellitus: a pilot study

**DOI:** 10.1186/1757-4749-5-14

**Published:** 2013-06-13

**Authors:** Saleh A Naser, Saisathya Thanigachalam, C Thomas Dow, Michael T Collins

**Affiliations:** 1 Burnett School of Biomedical Sciences, College of Medicine, University of Central Florida, Orlando, Florida 32816, USA; 2 Department of Ophthalmology and Visual Sciences, University of Wisconsin, Madison, WI, USA; 3 Department of Pathobiological Sciences, School of Veterinary Medicine, University of Wisconsin, Madison, WI, USA

**Keywords:** Type 1 diabetes, *Mycobacterium avium* subspecies *paratuberculosis*, Hsp65, GAD65, Crohn’s disease

## Abstract

**Background:**

Although the etiology of Type 1 Diabetes mellitus (T1DM) has not been determined, genetic polymorphism in key genes, including *SLC11A1*, and association with *Mycobacterium avium* subspecies *paratuberculosis* (MAP) have been reported. We hypothesize that molecular mimicry between MAP Heat shock protein 65 K (Hsp65) and human Glutamic Acid Decarboxylase 65 K (GAD65) may be the trigger leading to autoimmune destruction of beta cells in patients exposed to MAP.

**Method:**

Peptide sequences of MAP Hsp65 and human GAD65 were investigated for amino acid sequence homology and cross reactivity. A total of 18 blood samples from T1DM and controls were evaluated for the presence of MAP.

**Results:**

Peptide BLAST analysis revealed a 44% overall identity between MAP Hsp65 and GAD65 with 75% positives in a 16 amino acid region. PyMOL 3D-structural analyses identified the same 16 amino acid region as a potential epitope for antibody binding. Preliminary data suggests a cross reactivity between MAP Hsp65, and a healthy rat pancreatic tissue homogenate against plasma from T1DM patients and rabbit polyclonal anti-MAP IgG. Long-term culture of human blood resulted MAP detection in 3/10 T1DM and 4/8 controls whereas MAP IgG was detected in 5/10 T1DM samples and 3/8 non-diabetic controls.

**Conclusion:**

The high degree of homology between GAD65 and MAP Hsp65 in an antigenic peptide region supports a possible mycobacterial role in triggering autoimmune destruction of pancreatic cells in T1DM. Reactivity of T1DM patient sera with MAP Hsp65 supports this finding. Culture of MAP from the blood of T1DM patients is intriguing. Overall, the preliminary data are mixed and do not exclude a possible role for MAP in T1DM pathogenesis. A larger study including well-characterized controls is needed to investigate the intriguing question of whether MAP is associated with T1DM or not?

## Findings

### Background

Type 1 diabetes mellitus (T1DM) is a chronic disease in which the insulin producing beta cells of the pancreas are selectively destroyed by T lymphocyte infiltration [1-3]. It is the second most common chronic childhood disease and accounts for 5 to 10% of all diabetic cases [1]. According to the American Diabetes Association, T1DM can be broadly classified into two forms: (a) type 1A is usually characterized by the presence of antibodies against host proteins such as insulin, heat shock protein 60, insulinoma associated proteins (IA-2) and glutamic acid decarboxylase (GAD65) and (b) type 1B which is less frequent and has no known cause. GAD is a 65 KDa enzyme that catalyzes the synthesis of gamma-amino butyric acid (GABA) by α-decarboxylation of L-glutamic acid [4,5]. It is expressed in several tissues including pancreatic islets where it is considered to be one of the major auto-antigens involved in the pathogenesis of T1DM. In fact, antibodies to GAD65 are routinely used for diagnosis of the disease [4]. Persistence of hyperglycemia for prolonged periods leads to serious sequelae such as diabetic nephropathy, diabetic neuropathy, diabetic retinopathy, cardiovascular ailments and stroke [1]. Insulin replacement therapy is the only symptomatic treatment available for T1DM [1,6].

Genetic susceptibility for T1DM has been proposed. Inherited susceptibility factors mainly include the HLA genotypes *DR* and *DQ* and insulin-dependent diabetes mellitus (*IDDM*) genes [1,7,8]. Though genetic predisposition plays a major role in pathogenesis of T1DM, concordance studies on monozygotic twins, the rising incidence over the last 50 years and migrant studies suggest that exogenous factors also pay an important role [9]. Viruses, such as enterovirus and coxsackie, and more recently bacteria such as *Mycobacterium avium* subspecies *paratuberculosis* (MAP) have been proposed as possible triggers for the autoimmune response [10,11]. Dietary factors associated with T1DM include consumption of cow’s milk, wheat protein and lack of vitamin D [7,12]. The pathophysiology of T1DM is studied using animal models such as non-obese diabetic (NOD) mouse and Bio Breeding (BB) rat, but the trigger for autoimmune-mediated tissue damage remains unknown [13].

MAP has been proposed as a trigger for many autoimmune diseases such as multiple sclerosis, autoimmune thyroiditis, rheumatoid arthritis and autoimmune diabetes [14]. There is increasing evidence of shared genetic susceptibility between T1DM and mycobacterial infections which supports the role of MAP as a possible trigger [6,15,16]. One example is the *SLC11A1* (Solute carrier 11a1) gene which encodes an integral membrane protein of the lysosomes of monocytes and macrophages [17]. During infection, the *SLC11A1* causes acidification of phagosomes which helps protect the host against infection. Mutations in *SLC11A1* lead to malfunction of the protein, hampering phagosome acidification, leading to a more hospitable environment for bacterial survival and replication. Sechi et al. reported that polymorphisms in *SLC11A1* gene were associated with MAP infection in T1DM patients in Sardinia [17]. The same group also reported an elevated antibody response to MAP-specific proteins such as MAP3733c and MAP3738c in Sardinian T1DM patients [6,18].

Epitope homology between human antigens and MAP proteins may serve as a trigger for activation of autoimmunity [14,19,20]. Mycobacterial Hsp65 has been implicated in autoimmune diseases such as rheumatoid arthritis, autoimmune hepatitis, Kawasaki disease, scleroderma, Behcet disease and Takayasu’s arteritis [14]. MAP Hsp65 encodes 541 amino acids and Mtb Hsp65 encodes for 540 amino acids with both expressing an estimated 65KDa protein (http://www.uniprot.org/). We hypothesize that molecular mimicry between MAP Hsp65 and human GAD65 might trigger an autoimmune reaction targeting beta cells in pancreatic islets leading to insulin deficiency and T1DM [9,10,14].

## Results

### Bioinformatic analyses of sequence homology between MAP Hsp65 and GAD65

**Figure 1:**
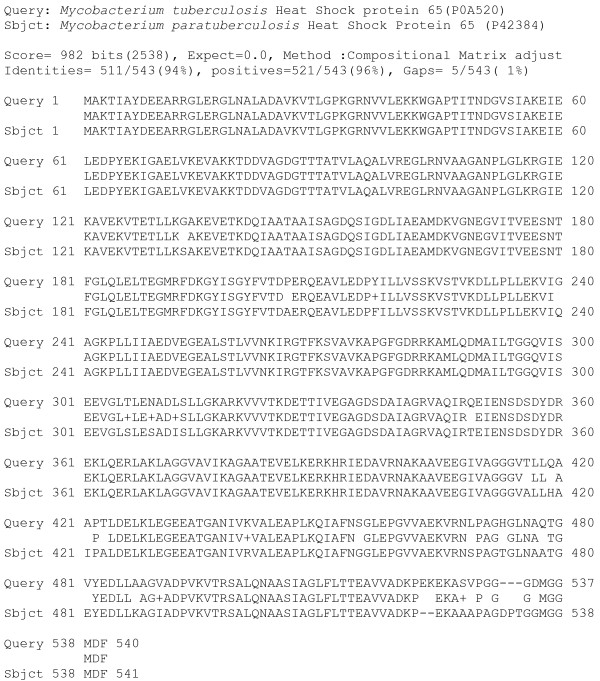
**BLAST analysis between Mtb Hsp65 and MAP Hsp65 peptide sequences.** Query peptide sequence is *Mycobacterium tuberculosis* Heat Shock protein 65(P0A520). Subject peptide sequence is *Mycobacterium paratuberculosis* Heat Shock Protein 65 (P42384).

**Figure 2:**
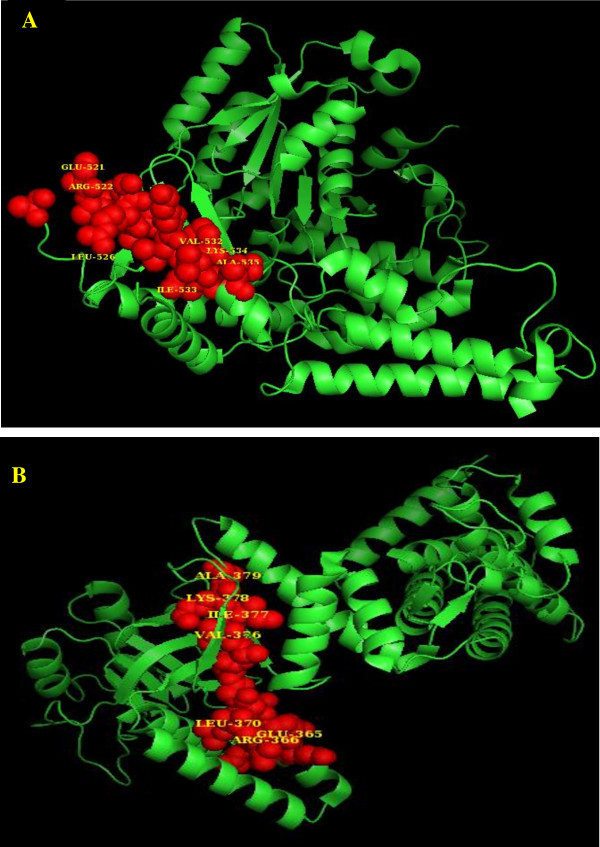
**PyMOL structural analysis of Mtb *Hsp65 *and human *GAD65. *A**: Protein structure of human GAD65 PDB ID: 2OKK [23]**B**: Protein structure of Mtb Hsp65 PDB ID:3RTK [24]. Highlighted regions represent the amino acids that were identical between GAD65 and MAP Hsp65 by BLAST analysis. Mtb Hsp65 and MAP Hsp65 are 100% homologous in this 16 amino acid region.

Although Mtb Hsp65 including its 3D-conformational structure is well characterized, MAP Hsp65 is not [21]. BLAST analysis of the Mtb Hsp65 with MAP Hsp65 peptide sequences revealed 96% positive amino acids with 94% amino acid identity (Figure 1). More importantly, a 44% identity was observed between MAP Hsp65 and human GAD65, with 75% positive amino acids in a specific 16 amino acid region (Table 1). The homology between Mtb Hsp65 and MAP Hsp65 within the 16 amino acid region was 100% (Table 1). The PyMOL visualization tool was used to localize and identify the same 16 amino acids peptide region in protein sequences of Mtb Hsp65 and human GAD65. As shown in Figure 2, PyMOL analysis localized the 16 amino acid epitope in human GAD65, and identified it as antigenic site targeted by autoantibodies in T1DM [22].

**Table 1:** **BLAST analysis between MAP *Hsp65***, **Mtb *Hsp65 *and *GAD65 *peptide sequences**

**Protein**^**a**^	**Amino acid region**	**Amino acid sequence**	**Amino acid sequence**
GAD65	520-535	EERMSRLSKVAPVIKA	
		+ER + ++L+ VIKA	75%
MAP Hsp65	364-379	QERLAKLAGGVAVIKA	
		QERLAKLAGGVAVIKA	100%
Mtb Hsp65	364-379	QERLAKLAGGVAVIKA	

a: Sequence identity between MAP Hsp65 and Mtb Hsp65 is 100% within this 16 amino acid region and 75% between GAD65 and both mycobacterial protein.

Abbreviations: *GAD65* Glutamic Acid Decarboxylase, *MAP Mycobacterium avium* subspecies *paratuberculosis*, *Mtb Mycobacterium tuberculosis*, *Hsp65* Heat shock protein of 65 kDa.

### Evaluation of antigenic cross reactivity between Mtb Hsp65, MAP Hsp65 and GAD65

**Figure 3:**
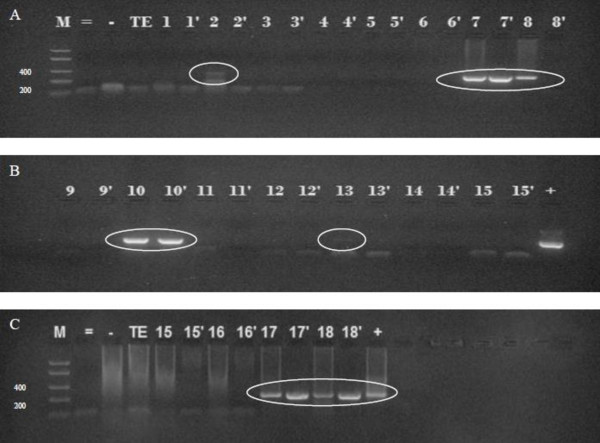
**Nested PCR detection of MAP DNA in blood culture.** Two rounds of nested PCR consisting of first set using P90/91 oligonucleotide primers and second round set using AV1/AV2 oligonucleotides primers. PCR product of 298 base pair indicates positive result for MAP. M: Molecular weight marker in base pair. =: Negative control for second round of PCR. -: Negative control for first round of PCR. TE: Negative control for DNA extraction. +: DNA template from MAP strain UCF4 as a positive control. Lanes 1 to 18: correspond with the code of each blood sample used in this study. Codes with a prime (‘) indicate that 1 uL of PCR product from P90/91 PCR set was used as template for the second AV1/AV2 round of PCR whereas codes without prime indicate that 23 uL of PCR product from P90/91 PCR set was used as template.

Blood from patient TD8 (subject with T1DM) was positive for MAP by culture and nested PCR (lane 8 Figure 3 and Table 2) and also for anti-MAP IgG (Table 2). Preliminary data from immunoblot analysis suggests a cross reactivity between Hsp65 from Mtb strain 25177, *M*. *avium* strain 25291, clinical MAP isolate, *E*. *coli* recombinant clone of MAP Hsp65 designated pmptb20, and pancreatic tissue lysate from a healthy rat using rabbit polyclonal anti-MAP IgG (data not shown). Furthermore, plasma from TD8 reacted strongly with MAP proteins (Table 2).

**Table 2:** Demographic information and MAP results for clinical samples used in this study

**Presence of MAP**^**a**^						
**Code**	**Sex**	**Diagnosis**^**b**^	**Source**^**c **^**specimen**	**Direct PCR**^**d**^	**Culture**^**e**^	**Anti-MAP IgG**^**f**^
TD 3	F	T1DM	UF	Whole Blood negative	negative	**positive**
TD 4	M	T1DM	UF	Whole Blood negative	negative	**positive**
TD 5	F	T1DM	UF	Whole Blood negative	negative	**positive**
TD 6	M	T1DM	UF	Whole Blood negative	negative	**positive**
TD 7	M	T1DM	UF	Whole Blood negative	**positive**	negative
TD 8	F	T1DM	UF	Whole Blood negative	**positive**	**positive**
TD 9	M	T1DM	UF	Whole Blood negative	negative	negative
TD 14	F	T1DM	UF	Whole Blood negative	negative	negative
TD 15	M	T1DM	UF	Whole Blood negative	negative	negative
TD 18	F	T1DM	UF	Whole Blood negative	**positive**	negative
TD 1	M	T2DM	UF	Whole Blood negative	negative	negative
TD 2	M	T2DM	UF	Whole Blood negative	**positive**	negative
TD 10	F	CONTROL	UF	Whole Blood negative	**positive**	negative
TD 11	F	CONTROL	UF	Whole Blood negative	negative	negative
TD 12	F	CONTROL	UF	Whole Blood negative	negative	negative
TD 13	F	CONTROL	UF	Whole Blood negative	negative	**positive**
TD 16	F	CONTROL	UF	Whole Blood negative	negative	negative
TD 17	M	CONTROL	UF	Whole Blood negative	**positive**	**positive**

a: *MAPMycobacterium avium* subspecies *paratuberculosis*.

b: *T1DM* Type I Diabetes Mellitus, *T2DM* Type two Diabetes Mellitus.

c: *UF* University of Florida.

d: direct PCR: nested PCR was performed on DNA extracts from uncultured peripheral leukocytes.

e: culture: nested PCR was performed on DNA extracts from 6 months old culture of peripheral leukocytes.

f: anti-MAP IgG: IDEXX serologic kit was used to measure anti-MAP IgG titer.

### Detection of MAP DNA

Human blood samples were all negative for MAP DNA in uncultured buffy coat preparations from all subjects using direct nested IS*900* PCR (Table 2). Following 6 months incubation, culture aliquots subjected to DNA extraction and nested IS*900* PCR revealed that MAP DNA in 3/10 T1DM, 1/2 T2DM and 3/6 controls. As shown in Figure 3, MAP DNA was detected in lanes 7, 8 and 18 (T1DM), lane 2 (T2DM) and lanes 10, 13 and 17 (controls). Table 3 summarizes the PCR results in all samples.

**Table 3:** Summary of results of clinical analysis of human samples

	**MAP**	**Anti**-**MAP IgG**
T1DM	3/10 (30%)	5/10 (50%)
T2DM	1/2 (50%)	1/2 (50%)
CONTROL	3/6 (50%)	2/6 (33.33%)

### Detection of MAP IgG antibodies

A commercial ELISA kit (IDEXX Laboratories, Inc., Westbrook, ME) used for diagnosis of Paratuberculosis in cattle was adapted for use on humans to measure anti-MAP IgG in plasma from all samples used in this study. The analysis was repeated twice and the average of all attempts was reported. As shown in Table 2 and Table 3, MAP antibodies were detected in 3/8 (37.5%) controls compared to 5/10 (50%) T1DM samples.

## Discussion

Association of MAP with T1DM has been reported exclusively in Sardinia, Italy and by Sechi et al. [6,15-18,25,26]. In the present study, we explored the possibility of MAP Hsp65 as a possible trigger for auto-destruction of beta cells through cross-reactivity with GAD65. BLAST analysis between MAP Hsp65 and human GAD65 revealed a 16 amino acid region with 75% positive amino acid sequence (Table 1). Interestingly, the same 16 amino acid region in GAD65 was previously suggested to be a potential antigenic binding site leading to auto destruction of pancreatic beta cells [22]. Since the PyMOL 3D structure of MAP Hsp65 is not available, the available peptide sequence of the closely related Mtb Hsp65 was used. In fact, the two heat-shock proteins have an overall amino acid identity of 94%, and 100% identity in the specific 16 amino acid region in question (Table 1). Figure 2 suggests that the 16 amino acid site localized on the 3D structure of MAP Hsp65 may provoke antibodies following MAP infection. The anti-MAP Hsp65 antibodies may then bind to the GAD65 epitope on the surface of Beta cell leading to their destruction. Preliminary immunohistochemistry data supports this hypothesis.

Detection of anti-MAP IgG in plasma from T1DM subjects (Table 3) is significant. More importantly is the detection of MAP DNA in blood culture obtained from T1DM subjects despite the fastidious nature of MAP. The detection of MAP in one of the two type 2 diabetes patients may be the results of disease misdiagnosis or the possibility of co-infection with other MAP-associated diseases. The same argument may be made for detection of MAP in uncharacterized controls.

The data partially corroborate the findings of Sechi et al. [6,15-18,25,26] and indicate that further study is needed. A larger study which includes well characterized controls is needed to appropriately evaluate a possible role of MAP in T1DM in other geographic regions. Molecular mimicry causing cross reactivity between MAP Hsp65 and human GAD65 may play a role in the destruction of pancreatic beta cells and development of T1DM.

## Materials and methods

### Bioinformatics analysis

Protein BLAST analysis was initialized to determine the sequence homology between peptide sequences of MAP Hsp65 and human GAD 65 (http://blast.ncbi.nlm.nih.gov/Blast.cgi). The peptide sequences of the proteins for the analysis were retrieved from Uniprot protein database (http://www.uniprot.org/). Predicted protein structures for PyMOL analysis were retrieved from the protein data bank (http://www.rcsb.org/pdb/home/home.do) and were used to localize the homologous sequences of the proteins in their 3D conformation.

### Evaluation of cross reactivity between Mtb Hsp65, MAP Hsp65 and GAD 65

#### Immunoblot analysis

Immunoblot analysis was used to evaluate the cross reactivity between MAP Hsp65 and human GAD65 following standard method [21].

#### Clinical samples

Blood samples were collected from 18 subjects. The demographic information including gender and diagnosis are listed in Table 2. Ten samples were collected from subjects with T1DM, 2 from subjects with Type 2 diabetes mellitus (T2DM) and from 6 non-diabetic controls. All subjects were free from inflammatory bowel disease. Informed consent was obtained from subjects in accordance with the Institutional Review Board (IRB) regulations. K_2_ EDTA-blood samples were collected and coded to mask patient identity and disease status.

#### Isolation of peripheral leukocytes

The K_2_ EDTA-blood tubes were transported to the laboratory cold within 24 hours. The blood was processed by centrifugation at 3000 × g for 10 minutes at room temperature. A volume of 1 mL of plasma was transferred to a new sterile tube and stored at −70°C for future testing for anti-MAP IgG, anti-GAD IgG, The leukocyte containing buffy coat layer was carefully transferred to a new sterile tube. Leukocytes were then mixed with two volumes of red blood cell lysis buffer (Roche Applied Sciences, IN, USA). The hemolyzed samples were then centrifuged at 2500 × g rpm for 5 minutes at room temperature. The supernatant was discarded and the leukocyte pellet was either inoculated into culture media or stored at −20°C for further use [27].

#### Culture of peripheral leukocytes

The purified buffy coat cells were inoculated into Mycobacterial Growth Indicator Tubes (MGIT) ParaTB Medium™ (BD Diagnostic Systems, Sparks, MD). Each tube of MGIT ParaTB medium was supplemented with 800 μl of OADC supplement (BD Diagnostic Systems, Sparks, MD) and 500 μl of egg yolk enrichment (BD Diagnostic Systems, Sparks, MD). All tubes were incubated at 37°. Following 6 months incubation, culture aliquots were subjected to DNA extraction and nested IS*900* PCR analysis.

#### DNA extraction from peripheral leukocytes

The isolated uncultured leukocytes were re-suspended in Tris EDTA (TE) buffer and then subjected to genomic DNA extraction following Naser et al. method [27].

#### Nested PCR for MAP DNA detection

DNA extracts were subjected to nested IS*900* PCR analysis [27]. The first round of PCR reaction mixture consists of 25 μl of Master Mix (50units/ml- *Taq*DNA polymerase, 400 μM-each dATP, dGTP, dCTP, dTTP and 3 mM MgCl_2;_ Promega, Madison, WI, USA), 1 μl of each primer (Forward primer: P90-5′-GTTCGGGGCCGTCGCTTAGG-3′, Reverse primer: P91-5′-GAGGTCGATCGCCCACGTGA-3′) and 23 μl of DNA template. In the second round of PCR reaction there was two sets of reactions which differed by the volume of the DNA template used. First set of the reaction mixture consisted of 25 μl of Master Mix, 1 μl of each primer (Forward primer: AV1-5′ATGTGGTTGCTGTGTTGGATGG’3, Reverse primer: AV2-5′CCGCCGCAATCAACTCCAG 3′) and 23 μl of DNA template which is the PCR product of the first round of reaction. The second set of the reaction mixture consists of the same except that 1 μl of PCR product from the first reaction was used as a template and the total volume was made to 50 μl by addition of nuclease free water. The cycle conditions of the primary reaction were: 95°C for 5 min, 35 cycles of 95°C for 1 min, 58°C for 1.5 min, 72°C for 1.5 min and final extension of 10 min at 72°C. The cycle conditions of the secondary reaction were: 95°C for 5 min, 35 cycles of 95°C for 0.5 min, 60°C for 0.5 min, 72°C for 1 min and final extension of 10 min at 72°C. The PCR product was analyzed on 2% agarose gel.

#### Detection of MAP IgG antibodies

The IDEXX MAP antibody test kit (IDEXX Laboratories, Westbrook, ME, USA) was modified for measurement of anti-MAP IgG levels in human samples. Plasma was diluted fifty fold (1:50) with the sample diluents. HRP-conjugated rabbit anti-human IgG was used at a final concentration of 1:100. Assays were performed in duplicate and reported as described by the manufacturer.

## Abbreviations

MAP: *Mycobactrium avium* subspecies *paratuberculosis*; T1DM: Type I diabetes mellitus; Hsp65: Heat shock protein 65 K; GAD65: Glutamic acid decarboxylase 65 K; GABA: Gamma-amino butyric acid; NOD: Non-obese diabetic; BB: Bio breeding; IA-2: Insulinoma associated proteins; IDDM: Insulin-dependent diabetes mellitus; Mtb: Mycobacterium tuberculosis; MGIT: Mycobacterial growth indicator tubes; IRB: Institutional review board.

## Competing interests

The authors declare that they have no competing interests.

## Authors’ contributions

SAN coordinated and supervised the entire project including daily experiments and led in writing of the manuscript. ST, a graduate student who performed all the experiments and assisted in writing the manuscript. CTD and MTC were instrumental in formulating the project goals and designing the experiments and equally helped to draft the manuscript. All authors read and approved the final manuscript.
